# Mitochondria-Targeted Delivery Strategy of Dual-Loaded Liposomes for Alzheimer’s Disease Therapy

**DOI:** 10.3390/ijms241310494

**Published:** 2023-06-22

**Authors:** Leysan Vasileva, Gulnara Gaynanova, Farida Valeeva, Grigory Belyaev, Irina Zueva, Kseniya Bushmeleva, Guzel Sibgatullina, Dmitry Samigullin, Alexandra Vyshtakalyuk, Konstantin Petrov, Lucia Zakharova, Oleg Sinyashin

**Affiliations:** 1 Arbuzov Institute of Organic and Physical Chemistry, FRC Kazan Scientific Center, Russian Academy of Sciences, 8 Arbuzov Str., 420088 Kazan, Russia; 2 Kazan Institute of Biochemistry and Biophysics, FRC Kazan Scientific Center, Russian Academy of Sciences, 2/31 Lobachevsky Str., 420111 Kazan, Russia; 3 Institute for Radio-Electronics and Telecommunications, Kazan National Research Technical University Named after A.N. Tupolev-KAI, 10 K. Marx St., 420111 Kazan, Russia

**Keywords:** liposome, surfactant, α-tocopherol, donepezil hydrochloride, Alzheimer’s disease, mitochondria

## Abstract

Liposomes modified with tetradecyltriphenylphosphonium bromide with dual loading of α-tocopherol and donepezil hydrochloride were successfully designed for intranasal administration. Physicochemical characteristics of cationic liposomes such as the hydrodynamic diameter, zeta potential, and polydispersity index were within the range from 105 to 115 nm, from +10 to +23 mV, and from 0.1 to 0.2, respectively. In vitro release curves of donepezil hydrochloride were analyzed using the Korsmeyer–Peppas, Higuchi, First-Order, and Zero-Order kinetic models. Nanocontainers modified with cationic surfactant statistically better penetrate into the mitochondria of rat motoneurons. Imaging of rat brain slices revealed the penetration of nanocarriers into the brain. Experiments on transgenic mice with an Alzheimer’s disease model (APP/PS1) demonstrated that the intranasal administration of liposomes within 21 days resulted in enhanced learning abilities and a reduction in the formation rate of Aβ plaques in the entorhinal cortex and hippocampus of the brain.

## 1. Introduction

Alzheimer’s disease (AD) has significant clinical, social, and economic consequences for society [1,2]. The number of newly diagnosed AD cases is expected to grow further owing to the increase in life expectancy and in the accuracy of diagnostic procedures, such as neuropsychological tests, computed tomography, and magnetic resonance imaging [3]. AD manifests mainly in sporadic form, and only 10% of cases are a result of genetic pathology [4,5,6]. There is currently no cure for AD treatment. All available medicines provide symptomatic relief with some improvement in the quality of life for patients with a mild to moderate disease form. A lack of consensus on the pathogenesis and etiology of AD is the reason for this ineffective therapy. This, in turn, makes it impossible to separate causes from consequences.

There are several established hypotheses that explain the pathogenesis of AD. The so-called “cholinergic hypothesis” is associated with a decrease in the synthesis of the neurotransmitter acetylcholine. Thus, cholinesterase inhibitors such as donepezil, rivastigmine, and galantamine are currently used for symptomatic therapy of AD [7,8]. The amyloid beta (Aβ) hypothesis is based on the abnormal hydrolysis of amyloid precursor protein, leading to the extracellular accumulation of the amyloid peptides Aβ_(1→40)_ and Aβ_(1→42)_, which gradually aggregate into neurotoxic plaques [9,10]. The tau hypothesis is associated with the observation of the formation of intracellular neurofibrillary tangles based on hyperphosphorylated tau protein [11]. However, research based on these three classical theories has not led to the development of effective drugs for AD treatment. In addition, the frequency of side effects and increasing dosages of medications have prompted scientists to search for new potential therapeutic targets. In light of the main goal of our study, the hypothesis of mitochondrial dysfunction is a relevant topic [12,13,14,15]. The dysfunction of neuronal cell mitochondria is currently considered to be a marker of the early stage of AD, appearing 10–20 years before clinical manifestations of the disease. The appearance of reactive oxygen species, disruption of the mitochondrial structure, and the development of oxidative stress, followed by the start of apoptosis, are the result of mitochondrial dysfunction [16,17]. It is important to note that mitochondrial dysfunction leads to additional accumulation of amyloid beta , which, in turn, disrupts the functioning of mitochondria, i.e., a so-called “vicious circle” is formed, which is very difficult to “break” [18]. Antioxidants such as vitamin E, vitamin C, CoQ10, curcumin, quercetin, resveratrol, caffeine, and α-lipoic acid have been tested for AD therapy [19,20]. Another possible reason for AD development is the use of devices based on electromagnetic radiation, such as phones, wireless internet, bluetooth devices, ovens, radars, and laptops [21,22]. Microwave-induced neurotransmitter damage delays the signaling process, causing harmful damage to the body.

Interest in the search for medicines that act simultaneously on several molecular targets, i.e., “multitarget drugs”, is explained by the multifactorial nature of AD [23,24,25,26]. On the one hand, combination therapy can be implemented through the targeted synthesis of new compounds that combine different types of activity [27,28,29,30]. However, the search for and creation of new drugs is a long and expensive path. Therefore, it is interesting to reveal new facets of already existing drugs either in a simple combination of two drugs with each other or by encapsulating them in nanocontainers [1,31,32,33]. Within the scope of the second strategy, our attention was drawn to liposomes that can encapsulate both hydrophilic and hydrophobic substrates [34,35,36,37,38,39,40] and allow for combination therapy of AD. In addition, it should be noted that liposomes have other advantages, including high biocompatibility, bioavailability, the ability to overcome biological barriers, and the protection of encapsulated substrates from premature degradation [41,42,43,44]. Of the three routes of administration of nanoformulated drugs for AD therapy—oral, transdermal, and intranasal—the latter is considered as the most promising [45]. The use of liposomes for intranasal drug delivery has been documented in numerous examples [46,47,48,49].

**Figure 1:**
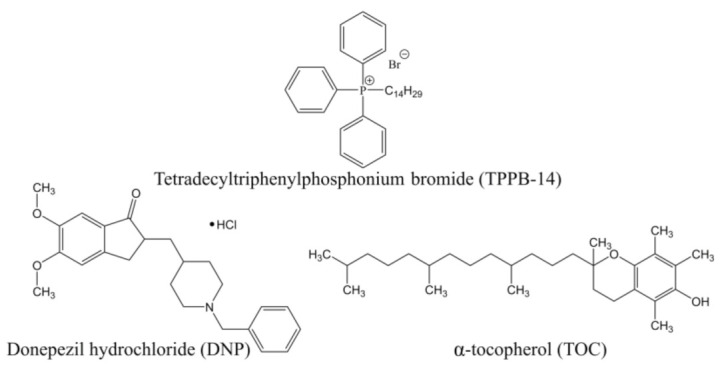
Chemical structures of liposome components.

Therefore, the aim of this work was to develop cationic liposomes consisting of soy phosphatidylcholine (PC), cholesterol (Chol), and tetradecyltriphenylphosphonium bromide (TPPB-14), loaded with antioxidant α-tocopherol (TOC) in the lipid bilayer and donepezil hydrochloride (DNP) in the water core (Figure 1) for intranasal administration. After the optimization of the liposomal formulation, in vitro studies on the colocalization of modified liposomes with the mitochondria of neuronal cells were carried out. Then, cationic liposomes were tested in vivo as a dosage form for the therapy of transgenic mice with a model of AD (APP/PS1). The in vivo experiment was carried out in three stages: (1) a behavioral test for the recognition of a novel object; (2) a quantitative assessment of Aβ plaques in the brain; (3) a quantitative assessment of the immunoexpression intensity of synaptophysin, a molecular marker for the presynaptic vesicles.

## 2. Results

The objects of the present study were liposomes of classical composition (soy phosphatidylcholine and cholesterol), modified with a cationic surfactant with a triphenylphosphonium head group for dual loading of α-tocopherol (10%) (TOC) and donepezil hydrochloride (DNP). At the first stage, the influence of various lipid concentrations and the presence of TOC on the physicochemical characteristics of the formulations was evaluated. The primary goal in developing nanosized drug delivery systems is achieving controlled aggregate sizes (≈100 nm) with a low polydispersity index (PdI ≤ 0.25). This task can be successfully achieved using an extruder in the final stage of nanoparticle preparation. As can be seen from the data obtained by dynamic light scattering (DLS), the hydrodynamic diameter (D_h_) of all systems on the day of preparation was in the range of 103–115 nm, and the PdI was between 0.067 and 0.231 (Table 1). The stability of the systems was monitored by DLS at certain time intervals. It was found that the liposomes are stable for more than 5 months at 4 °C, after which changes in the determined DLS parameters (D_h_, PdI, and zeta potential (ζ)) were observed (Table S1).

**Figure 2:**
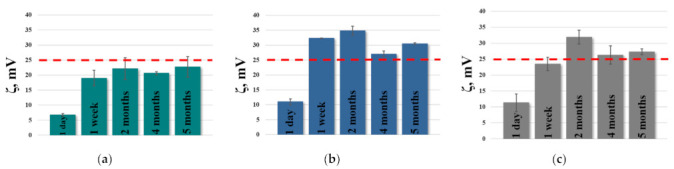
Diagram of changes in zeta potential of PC/Chol/TOC/TPPB-14 liposomes during storage. Liposome concentration is: (**a**) 15 mM; (**b**) 20 mM; (**c**) 30 mM.

The zeta potential is another important factor that determines the effectiveness of nanocarriers for intranasal delivery. It is known that cationic particles are retained longer on the nasal mucosa due to electrostatic interaction between positively charged nanoparticles and negatively charged mucin residues. Figure 2 shows the diagrams of changes in the zeta potential of PC/Chol/TOC/TPPB-14 liposomes (15 mM, 20 mM, and 30 mM) during storage. As can be seen, low zeta potential values on the first day of preparation were characteristic for all three systems with different lipid concentrations. The zeta potential in all cases was equilibrated within a week and remained approximately at the same level for up to 5 months of storage. It should be noted that the same trend was observed for cationic systems without TOC at different lipid concentrations. In each case, the maximum zeta potential of liposomes was observed in the second month of measurements (Table S1).

It is also worth noting that in the case of the system with 15 mM of lipid content, the zeta potential barely reaches +25 mV (Figure 2a), whereas in systems with 20 mM (Figure 2b) and 30 mM (Figure 2c) of lipid content, the zeta potential exceeds this mark (red dotted line) and reaches +35 and +32 mV, respectively.

**Figure 3:**
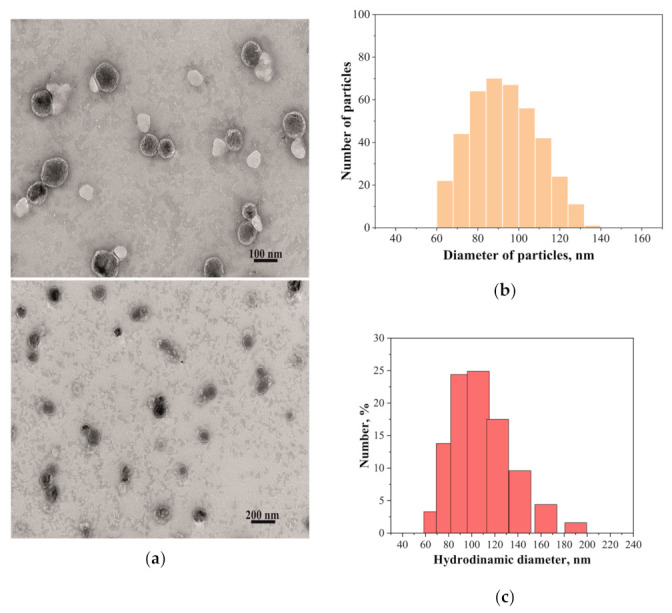
(**a**) TEM images of PC/Chol/TOC/TPPB-14 liposomes (20 mM); (**b**) size distribution of liposomes by number (TEM); (**c**) number averaged size distribution of particles (DLS), 25 °C.

Data from DLS, specifically the size and morphology of liposomes, were confirmed using transmission electron microscopy (TEM) for the PC/Chol/TOC/TPPB-14 system (20 mM). As seen in the microphotographs in Figure 3a, the liposomes had well-defined boundaries and a round shape with a size of around 90–100 nm. To compare the size of the liposomes obtained by TEM and DLS, all microphotographs were processed to measure the diameter of all particles in the field of view. The obtained results are presented as a diagram of the distribution of the number of particles by size in Figure 3b. It was found that the largest number of particles had a diameter of 80–110 nm, which is in good agreement with the DLS data. Figure 3c shows a diagram of the distribution of particles averaged by the number of particles, which qualitatively and quantitatively corresponds to the diagram obtained during the processing of the TEM results.

At the next stage, in order to select the most optimal system of modified liposomes, the cholinesterase inhibitor DNP was loaded into the liposomes. The efficiency of drug encapsulation is one of the fundamental criteria for selecting a lead system. It was determined for both DNP and TOC. For quantitative determination of the content of substrates in liposomes, their extinction coefficients were first determined (Figure S1). According to the calculations, there was no statistical difference in the encapsulation efficiency of TOC with varying lipid concentration (95 ± 1% for 15 mM, 96 ± 1% for 20 mM, and 30 mM), and the encapsulation efficiency of DNP slightly increased with incremental lipid concentration in the system (Figure S2). Based on the obtained data, a system with an average lipid content of 20 mM was chosen for further research. This is also due to the lower content of cationic surfactant in the system, compared to the 30 mM system, which reduces the risk of acute toxicity of liposomes. This system also had a higher zeta potential and stabilized more quickly (Figure 2b).

**Figure 4:**
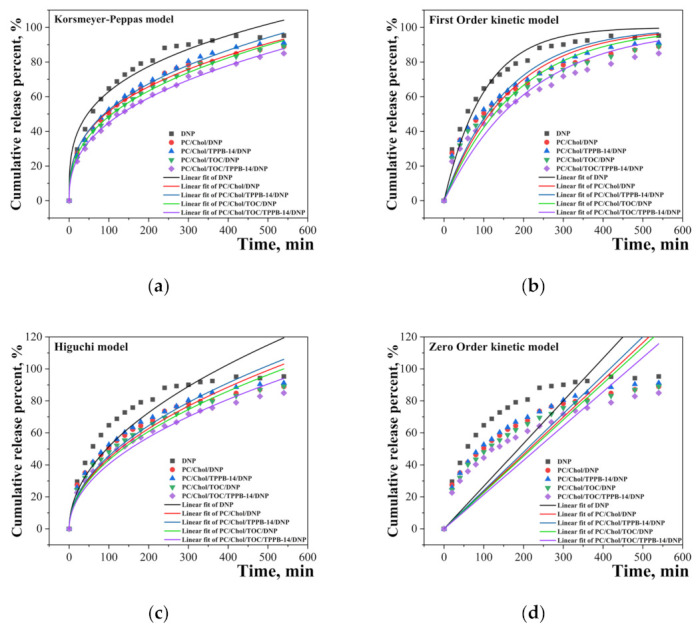
The release kinetic model fitting curves of DNP: (**a**) Korsmeyer–Peppas model; (**b**) First-Order kinetic model; (**c**) Higuchi model; (**d**) Zero-Order kinetic model. Total lipid concentration is 20 mM. Phosphate buffer (0.025 M), pH = 7.4, 37 °C.

The rate of DNP release from unmodified and modified liposomes was evaluated. The experiment was carried out on the example of a system with 20 mM of the lipid moiety. The main question that is interesting to answer is how the encapsulation of DNP and TOC affect the release rate of cholinesterase inhibitor. To trace the nature of DNP release, cumulative release percent versus time graphs were plotted (Figure 4). The absorption spectra of DNP are presented in Figure S3. It can be seen that the encapsulation of DNP in liposomes obviously leads to a decrease in its release rate from the dialysis bag. This phenomenon may have a positive effect when using the liposomal form of the drug in vivo, since a so-called prolongation of action is achieved, which can potentially allow “a reduction in the frequency of medication. It should be noted that the presence of TOC in the system enhances this effect in the case of modified liposomes. For a more detailed understanding of the DNP release, the resulting curves were processed using various kinetic models, namely, the Korsmeyer–Peppas, Higuchi, First-Order, and Zero-Order models (Figure 4).

The main parameters at this stage were the rate constant (k), the coefficient of determination (R^2^), and the diffusion release exponent indicating the mechanism of substrate release (n) (for the Korsmeyer–Peppas model). According to the results presented in Table 2, it becomes apparent that the Korsmeyer–Peppas model is the most suitable for describing the kinetics of DNP release from liposomes, since the R^2^ for liposomal systems exceeds 0.99. High R^2^ values have also been obtained for the Higuchi model, which predicts that drug release occurs via diffusion. This is confirmed by the values of the diffusion release exponent, determined by the Korsmeyer–Peppas model. The value of the exponent *n* ≤ 0.45 indicates that the drug substance is released according to Fick’s law or by the diffusion mechanism. For comparison, we also processed the DNP release curves in the initial part, namely, from 0 to 270 min (Figure S4). As shown in the graphs, in the initial section of the curves, the convergence of the experimental points with linear fits is much better. This is also confirmed by the values of the rate constant and the coefficient of determination (Table S2).

**Figure 5:**
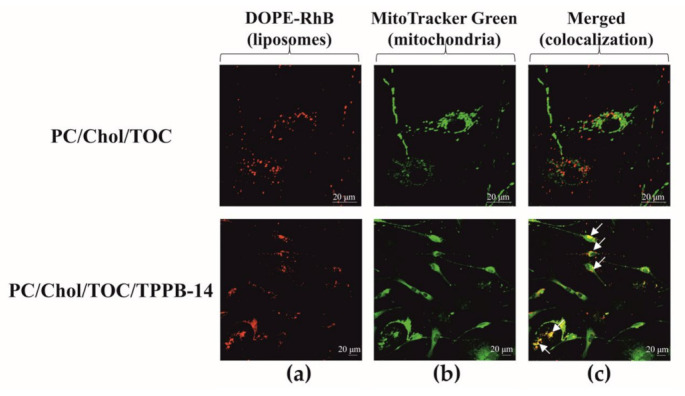
Analysis of colocalization of PC/Chol/TOC and PC/Chol/TOC/TPPB-14 (20 mM) liposomes with mitochondria of rat motoneurons: (**a**) cells are labeled with DOPE-RhB (liposomes); (**b**) cells are labeled with MitoTracker Green (mitochondria); (**c**) merged image with yellow color indicating colocalization of the two probes. Scale bar 20 μm.

Within this study, we relied on the mitochondrial cascade hypothesis of AD development and assessed the mitotropic activity of liposomes by incorporating TPPB-14 into their bilayer. The experiment was conducted on a culture of rat motoneurons using a confocal microscope. As seen in Figure 5, the modified PC/Chol/TOC/TPPB-14 liposomes penetrate the mitochondria of neuronal cells better than unmodified ones, as evidenced by the yellow fluorescence (overlap of fluorescence from two probes in column C). The calculated Pearson’s correlation coefficient values are 0.29 ± 0.01 and 0.46 ± 0.01 for liposomes composed of PC/Chol/TOC and PC/Chol/TOC/TPPB-14, respectively (Figure S5).

To determine the biological activity of the selected system in vitro, the antioxidant activity of liposomes with the antioxidant TOC was investigated using chemiluminescence analysis. Upon the introduction of antioxidants into the system, the number of radicals decreases, and with it, the intensity of chemiluminescence drops. The work by Lissi E.A. [50] describes an approach to measure total antioxidant capacity that takes into account this feature of the curves—the TAR (total antioxidant reactivity) method and the TRAP (total reactive antioxidant potential) method. It is believed that TRAP reflects the amount of antioxidant in the system, and the TAR method reflects its activity, i.e., the rate of interaction between the antioxidant and radicals. According to the results, the inclusion of TPPB-14 and TOC in the lipid bilayer increases the antioxidant activity of the system compared to individual TOC at the same concentration (Table 3). The degree of chemiluminescence quenching and the duration of the latent period in the presence of the tested samples are also presented in Figure S6.

**Figure 6:**
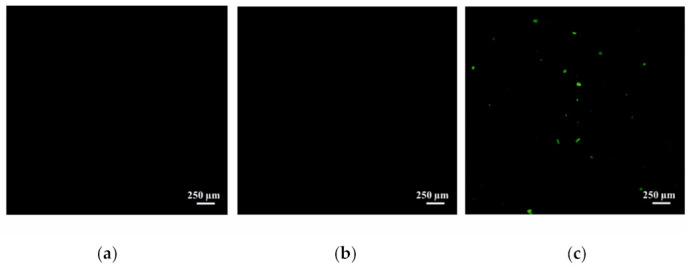
Cross-sections of rat brain: (**a**) control; (**b**) after administration of free RhB (0.5 mg/kg); (**c**) after intranasal administration of RhB (0.5 mg/kg) in PC/Chol/TPPB-14 (15 mM) liposomes. Scale bar 250 μm.

After confirming the stability of the liposomes, their capacity for dual loading of substrates, and their in vitro effectiveness, the study moved on to in vivo experiments. Firstly, the uptake of the liposomes into the brain via intranasal administration was tested. Rhodamine B (RhB) was selected as a visualizing agent and was encapsulated in the liposomes using a passive loading method. Free RhB and encapsulated RhB were administered intranasally at a dose of 0.5 mg/kg. Non-treated rats were used as a control. It was shown that the intranasal administration of cationic liposomes leads to the effective absorption of RhB in brain (Figure 6c), compared to the free form of the probe (Figure 6b) and the control group of rats (Figure 6a), presumably due to the high zeta potential. The identification of encapsulated RhB in the brain may indicate the ability of the investigated systems to bypass the blood–brain barrier.

**Figure 7:**
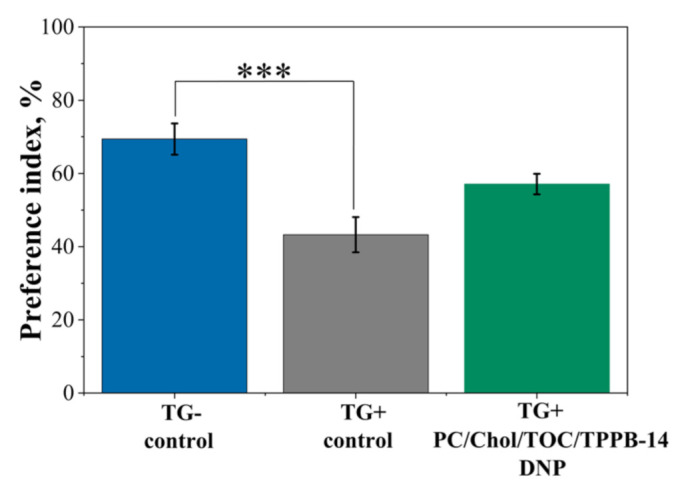
Distribution of the preference index of transgenic APP/PS1 mice for the novel object in the control group of wild-type mice (TG−, in the control group of transgenic mice (TG+), and in the group of transgenic mice (TG+) that intranasally received liposomes with TOC and DNP for 21 days. Data are presented as mean values ± SEM. ***—difference with regard to the control group of TG− mice is statistically significant at *p* ≤ 0.001. Statistical analysis was performed using ANOVA with Tukey’s post hoc test.

In the final stage of the study, PC/Chol/TOC/TPPB-14 liposomes (20 mM) loaded with both the cholinesterase inhibitor DNP and the antioxidant TOC were tested as a drug delivery system for the treatment of mice with an AD model. Since the goal of the experiment was to slow down the progression of the disease, therapy was started at an early stage when only the first signs of pathological changes were detected, corresponding to the age of mice at 6 months. The experiment was performed in two stages. The first stage was devoted to a behavioral test that allowed for the assessment of memory impairment (“the novel object recognition test”). Within 18 days before the test, and during the test (20 min prior to its start), the PC/Chol/TOC/TPPB-14/DNP liposomes were intranasally administered to the mice. It was shown that in the wild-type control group (TG−), the mice preferred the novel object over the familiar one with a probability of 69.4 ± 4.3% (Figure 7). In the case of the control group of transgenic mice (TG+), the preference for the novel object was significantly lower (43.3 ± 4.8%, *p* = 0.0006). At the same time, the group of transgenic mice that received intranasal administration of liposomes for 21 days showed an interest in the novel object with a probability of 57.1 ± 2.8%, which did not differ statistically (*p* = 0.077) from the control group of TG− wild-type mice (Figure 7). It is important to note that during the intranasal administration of liposomes loaded with DNP and TOC for 21 days, no side effects were observed. None of the mice showed any signs of behavioral changes or movement difficulty. Eating and drinking habits were normal.

**Figure 8:**
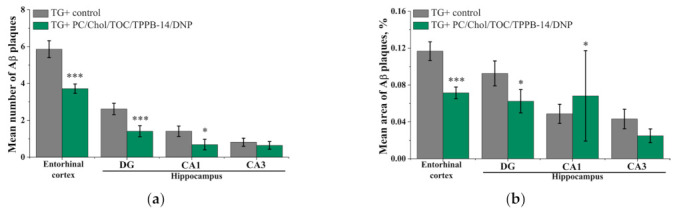
Mean number of Aβ plaques (**a**) and mean percentage of total area of Aβ plaques (**b**) in the entorhinal cortex and hippocampus of the brain in the control group of transgenic mice (TG+) and in the group of transgenic mice (TG+) receiving liposomes loaded with TOC and DNP. Data are presented as mean values ± SEM. *—difference with regard to the TG+ control group is statistically significant at *p* ≤ 0.05; *** at *p* ≤ 0.001. Statistical analysis was performed using the Mann–Whitney test.

At the second stage, a quantitative assessment of Aβ plaque formation and of the intensity of the synaptophysin immunoexpression in the brain of the control group of transgenic (TG+) mice and the group of transgenic (TG+) mice treated with PC/Chol/TOC/TPPB-14/DNP liposomes was carried out. It was shown that intranasal administration of PC/Chol/TOC/TPPB-14/DNP liposomes for 21 days significantly reduced the mean number of Aβ plaques and the mean percentage area of Aβ plaques in the hippocampus and entorhinal cortex of TG+ mice (Figure 8). Thus, the mean number of Aβ plaques in the entorhinal cortex decreased from 5.86 ± 0.46 in control TG+ mice to 3.72 ± 0.25 (*p* = 0.00013) in TG+ mice treated with liposomes (Figure 8a). The mean area of Aβ plaques decreased from 0.12 ± 0.01% to 0.07 ± 0.01% (*p* = 0.0008), respectively (Figure 8b). In the dentate gyrus (DG) of the hippocampus, the mean number of Aβ plaques decreased from 2.61 ± 0.31 to 1.41 ± 0.30 (*p* = 0.0008), and the mean area of Aβ plaques decreased from 0.09 ± 0.01% to 0.06 ± 0.01% (*p* = 0.03). In addition, a significant decrease in the number of Aβ plaques from 1.41 ± 0.28 to 0.68 ± 0.29 (*p* = 0.012) was observed in the area CA1 of the hippocampus after liposome administration (Figure 8a). Thus, intranasal administration of PC/Chol/TOC/TPPB-14/DNP liposomes for 21 days prevented memory impairment and slowed down the rate of Aβ plaque formation. Representative microphotographs of Aβ plaques in brain cross-sections of the entorhinal cortex and hippocampus of TG+ mice are shown in Figure S7.

**Figure 9:**
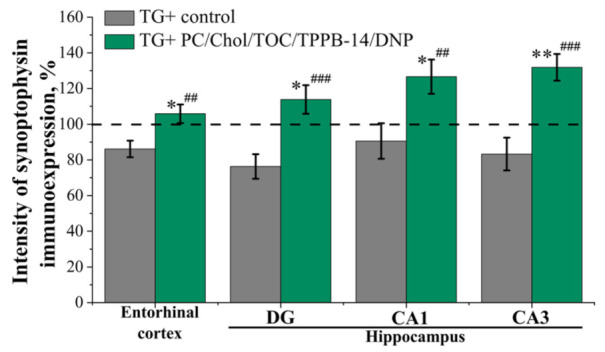
Intensity of synaptophysin immunoexpression in cross-sections of mice brain in entorhinal cortex and hippocampus in the control group of transgenic mice (TG−) and in the group of transgenic mice (TG+) receiving liposomes loaded with TOC and DNP. The dotted line shows the mean value of synaptophysin immunoexpression intensity in the control group of TG− mice. Data are presented as mean values ± SEM. *—significant difference from the control group of TG− mice at *p* ≤ 0.05; ** at *p* ≤ 0.01. ##—significant difference from the control group of TG+ mice at *p* ≤ 0.01; ### at *p* ≤ 0.001. Statistical analysis was performed using the Mann–Whitney test.

A quantitative assessment of the synaptophysin immunoexpression in the mice’s entorhinal cortex and hippocampus did not reveal significant changes in the intensity of immunoexpression between the control groups of TG− and TG+ mice (Figure 9). This absence of statistically significant differences is most likely explained by the early stage of pathology development in 6-month-old mice. The intranasal administration of PC/Chol/TOC/TPPB-14/DNP liposomes for 21 days led to a significant increase in the synaptophysin immunoexpression in the entorhinal cortex and hippocampus of transgenic mice (TG+) (Figure 9). Thus, in the entorhinal cortex, synaptophysin immunoexpression increased by 6% (*p* = 0.016) and 20% (*p* = 0.004) compared to the control TG− and TG+ mice, respectively. In the dentate gyrus (DG) of the hippocampus, immunoexpression intensity increased by 14% (*p* = 0.040) and 38% (*p* = 0.001), in the area CA1 by 27% (*p* = 0.016) and 36% (*p* = 0.009), and in the area CA3 by 32% (*p* = 0.004) and 49% (*p* = 0.0001) compared to control TG− and control TG+ mice, respectively.

## 3. Discussion

The aim of this study was to enhance the therapeutic effect of the cholinesterase inhibitor DNP by incorporating it into mitochondria-targeted cationic liposomes along with the antioxidant TOC. The cationic surfactant with a triphenylphosphonium head group was selected as the mitochondria-targeting agent, which, due to its amphiphilic nature, can easily integrate into the lipid bilayer of liposomes. Previously, our research group investigated the mitochondrial targeting of liposomes modified with triphenylphosphonium cationic amphiphiles with different lengths of hydrocarbon tails and at different ratios of amphiphiles/lipids in the case of cancer [51]. In addition to oncological diseases [52,53,54], the mitochondria-targeted drug delivery strategy is gaining more importance in the context of such disorders as retinal ischemia–reperfusion injury [55], cardiovascular diseases [56], stress-related neurodegenerative diseases [57,58,59], etc. It is worth noting that in the above cases and in many others, the key role in pathogenesis is played by dysregulation of the production of reactive oxygen species, for which the mitochondria of cells are responsible [60,61,62]. Such intense interest in mitochondria as targets for the treatment of various diseases is explained by the fact that mitochondria have a wide range of functions, such as the formation of cell energy units, the control of Ca^2+^ homeostasis, the signaling of reactive oxygen species, etc. According to one of the current hypotheses (the mitochondrial cascade hypothesis), mitochondrial dysfunction appears at the earliest stages of AD, which leads to oxidative stress in brain cells. Further, this can lead to additional accumulation of Aβ plaques, which, in turn, disrupts the functioning of mitochondria [63,64,65]. Therefore, the combination of traditional therapy for AD (inhibition of brain cholinesterase) with a mitochondria-targeted drug delivery strategy seems to be very attractive and promising. Based on previous experience, we focused on the tetradecyl homologue with a PC/amphiphile ratio of 50/1 and attempted to trace the influence of the lipid component of the systems, specifically its concentration (Table 4), on the physicochemical characteristics of the formulations.

It is known that the size distribution and polydispersity of liposomes can affect their physical stability, which, in turn, is influenced by the method of nanoparticle preparation, storage conditions, and zeta potential. For example, in [66,67], it was shown that liposomes exhibit better stability over time when stored at 4 °C compared to 25 °C and 37 °C. Many authors have also shown the critical role of the zeta potential of lipid nanoparticles in their stability during storage [68,69,70]. It has been established that a zeta potential of ≈30 mV is optimal for preventing their coagulation. The choice of production method is also an important step in the creation of lipid nanocontainers. For example, the method of ethanol injection with an incorrect selection of the component ratios can lead to the destruction of nanoparticles [71]. In addition, we assume that the uniformity of the size distribution of aggregates also affects the distribution of potential-carrying components in the bilayer (in our case, surfactant molecules), which brings us back to the role of the zeta potential in the stability of nanoparticles. From this point of view, methods based on the extrusion of liposomal dispersion are more likely to obtain a monodisperse system [72] than the ultrasonic method, for example [73]. Analysis of the data obtained using dynamic and electrophoretic light scattering (Table 1 and Table S1) allowed us to identify several patterns regarding the investigated formulations. (1) The addition of TPPB-14 to the system increases the PdI in all cases. Moreover, the higher the concentration of the lipid component (and the concentration of TPPB-14, respectively), the more pronounced this effect. This may be related to the fact that in the system with 20 mM lipids, the concentration of TPPB-14 is very close to the critical micelle concentration (by tensiometry), and in the system with 30 mM lipids, its concentration is slightly higher [74]. It is likely that surfactant molecules do not immediately incorporate into the lipid bilayer of the liposomes and remain in solution, contributing to the decrease in the monodispersity of the system. (2) Low zeta potential values of the liposomes on the day of preparation, which increase during storage for all systems, confirm the previous assumption, indicating that the investigated formulations require time to stabilize (approximately up to 1 week) (Figure 2). (3) Probably due to the gradual incorporation of TPPB-14 molecules into the lipid bilayer of liposomes, the PdI of unmodified liposomes increases, whereas in the case of modified systems, it decreases. (4) The inclusion of TOC in the lipid bilayer reduces the zeta potential of modified liposomes. These differences are statistically significant and were observed both on the first day of liposome preparation and after 5 months of storage (Table 1). Since TOC is a hydrophobic substrate, it can be assumed that the co-inclusion of the antioxidant and TPPB-14 in the lipid bilayer creates some competition, and TOC slightly hinders the incorporation of surfactants. However, this difference is not critical, as the zeta potential of modified liposomes in the presence of TOC remains above +25 mV (in the case of 20 mM and 30 mM) (Figure 2).

To confirm the size, morphology, and polydispersity of the liposomes, transmission electron microscopy (TEM) was used. The images clearly show that the liposomes have a rounded shape (Figure 3a) with an average diameter of 92 ± 17 nm (measured across all particles in the field of view). More detailed size distribution diagrams obtained using TEM and DLS can be seen in Figure 3b,c, respectively. It is important to note that DLS data reflect the diameter of the liposomes along with the solvent shell, and the sizes may be slightly larger than those determined by TEM. Given this fact, it can be concluded that the two methods used to determine the diameter of the investigated formulations are highly correlated with each other.

The proposed approach of dual drug loading in a nanocarrier is being actively developed now. Thus, there are some examples of combined delivery of drugs for AD therapy: (1) donepezil hydrochloride (or memantine hydrochloride)/insulin sensitizer in polycaprolactone-g-dextran-based polymer vesicles [75]; (2) metformin/romidepsin and rosiglitazone/vorinostat in poly(ethylene glycol)-poly(ε-caprolactone)-based polymer nanoparticles, additionally stabilized with poloxamer [76,77]; and (3) siRNA/rapamycin in a nanocarrier based on lectin and KLVFF peptides [78]. There is also an example of combining two drugs without nanocontainers—donepezil and memantine in Namzaric™ [79]. In the present work, it was extremely important to find out how much of both drugs, TOC and DNP, can be loaded into the lipid bilayer and the hydrophilic core of liposomes, respectively. There were two different methods for the determination of liposome encapsulation efficiency regarding DNP and TOC. For DNP, a common filtration/centrifugation technique was used to separate free and encapsulated substrate [80,81,82]. For TOC, the method of extraction of the unencapsulated substrate in ethanol was used [83,84]. It has been found that the encapsulation efficiency of liposomes toward TOC is independent of the total lipid concentration and is approximately 96%. In the case of DNP, some differences were found, namely, the higher the lipid content, the higher the EE (Figure S2). This dependence is quite explainable, since the higher the lipid concentration in the system, the more liposomes in the solution and, accordingly, “reservoirs” for the drug substance in the same volume. Based on the literature data, we can conclude that, in general, DNPs are characterized by fairly high encapsulation efficiency values: 93 ± 5.33% [85], 62.5 ± 0.6 [86], and 84.91 ± 3.31% [87], which was also shown in the present work.

For further investigations, we focused our attention only on one system, namely, modified liposomes with 20 mM of lipid content. Such a choice is based on the fact that this system showed the best zeta potential and sufficiently high encapsulation efficiency. In addition, a high concentration of surfactants in the system can increase the toxicity of liposomes toward biological systems in vitro and in vivo. Along with toxicity, drug release patterns are a very important characteristic of nanoscale drug delivery systems. In addition, the use of mathematical models to describe the kinetics of substrate release, and sometimes even to predict it, is becoming a classic tool in the development of dosage forms. The main and frequently used models in the literature are the Korsmeyer–Peppas, Higuchi, First-Order, and Zero-Order models [88,89,90], which were also applied in this work. First, as can be seen from Figure 4, the encapsulation of DNP in liposomes leads to a slowdown in the rate of its release from the dialysis bag, which is typical for nanoscale drug delivery systems [49,91]. This may be due to the fact that the substrate takes longer to cross the lipid bilayer first before being released from the dialysis bag. According to profiles presented in Figure 4, in the initial section of the curves, a rapid release of the substrate from the bag is observed, and the rate then slows down. It should be noted that the most prolonged release of DNP is observed from the PC/Chol/TOC/TPPB-14 system. Inclusion of TOC also slows down the release of the substrate, probably due to the denser packing of the lipid bilayer. This phenomenon is also confirmed by the data presented in Table 2, namely, the values of the rate constant (k) determined by different models. The PC/Chol/TOC/TPPB-14 system has the lowest release rate constant and the highest coefficient of determination (R^2^) in all models. The data obtained are important from the point of view of choosing the most optimal system for further experiments, as well as for the development of new formulations in the future. It also becomes clear that the Korsmeyer–Peppas and Higuchi models are the most suitable for describing the kinetics of DNP release, i.e., release occurs by diffusion mechanism [92]. It should be noted that the processing of the substrate release curves in their initial section confirms the data obtained for the entire curve (Figure S4 and Table S2). The absorption spectra of DNP at different time intervals for all systems are presented in Figure S3.

Prior to moving on to in vivo experiments, the mitotropic activity of liposomes was evaluated on a culture of rat motoneuron cells. The efficiency of the systems was evaluated based on two criteria: the presence of yellow staining, which appears when colocalizing the mitochondrial dye (i.e., mitochondria) and the fluorescent lipid (i.e., liposomes); and Pearson’s correlation coefficient, which reflects the relationship between the fluorescence intensity of two dyes. The closer the correlation coefficient value is to 1, the stronger the relationship between the two random variables, namely, the fluorescence intensities of the corresponding dyes. According to the results, modified liposomes indeed penetrate the mitochondria of neuronal cells better than unmodified ones, which is confirmed by microphotographs (Figure 5) and Pearson’s correlation coefficient (Figure S5).

As described earlier, the current study aimed to test the hypothesis that the combined use of an antioxidant with an cholinesterase inhibitor would affect the pathogenesis of AD. The antioxidant activity of free TOC and TOC in modified liposomes was determined using a luminol-induced chemiluminescence analysis in vitro. At this stage of the study, it was already clear that there was no need to test unmodified liposomes further, as they did not meet the required physicochemical characteristics, namely, the need for a cationic charge, and as a result, they did not possess mitotropic activity (Figure 5). The antioxidant activity was evaluated by two methods: TAR and TRAP. The TAR method was used to determine the degree of quenching of the luminescence intensity of peroxyl radicals, and the TRAP method measured the latency period of the peroxyl radical curve. It should be noted that the choice of 2,2′-azobis(2-amidinopropane) dihydrochloride (AAPH) as the source of radicals was not random, since the rate of decomposition of azo compounds is not affected by additives, which, together with several methods of assessment, increases the quality and purity of the experiment [93]. From the data presented in Table 3, it can be seen that free TOC has low antioxidant activity (36.4 ± 2.9%), despite being considered a strong antioxidant. This may be due to the hydrophobicity of TOC and the fact that the experiment is conducted in an aqueous solution, where TOC’s low solubility in such a medium may lead to rapid inactivation as indicated by the short latent period (slightly over 3.5 min) (Table 3). Similar assumptions were also made by the authors of [94], as TOC activity is usually determined in non-polar environments. Liposomes are ideal for solving this problem, as they can encapsulate both hydrophilic and hydrophobic substrates, increasing their solubility. Indeed, in the case of modified liposomes loaded with TOC, a longer latent period of interaction between radicals and the antioxidant system is observed (more than 8 h) (Figure S6) with an almost 100% reduction in chemiluminescence, possibly due to the protective action of liposomes toward TOC (Table 3).

After evaluating the physicochemical characteristics of liposomes and their efficacy in vitro and ex vivo, the study proceeded to in vivo experiments. The ability of cationic liposomes to be absorbed into the rat brain via intranasal administration was first tested. Visualization of the free Rhodamine B (RhB) and the liposome-incorporated RhB in brain tissue sections was carried out using a fluorescent microscope. According to the results presented in Figure 6, free RhB does not reach the brain at all, whereas in the case of modified liposomes, there is an effective delivery of the probe to the brain, as evidenced by the green fluorescence in Figure 6c. We hypothesize that this difference is due to the fact that modified liposomes are able to remain in the nasal mucosa for a longer period of time, primarily due to their high positive zeta potential. Similar assumptions have already been put forward by several research groups, but this mainly concerns polymeric aggregates [95,96,97]. The size of the carriers is also important for intranasal drug delivery. In [98], it was experimentally demonstrated that nanoparticles with a diameter <200 nm were better retained, and for longer periods of time, in the mucous membrane of the nasal cavity of rats, which makes it possible to increase the effectiveness of drug delivery to the brain.

Currently, a large number of research groups are working on the problem of increasing the effectiveness of DNP in the treatment of AD. These groups are mainly focused on finding an optimal delivery system and its route of administration [99]. In this regard, the intranasal route of administration appears to be an attractive choice for many researchers, who have demonstrated the effectiveness of DNP-loaded liposomes, SLNs, nanoemulsions, etc., compared to the free form of DNP [87,100,101,102,103,104]. It is worth noting that although there is evidence in the literature confirming the effectiveness of intranasal administration of DNP included in nanocarriers in vitro and in vivo, there are almost no in vivo results on animal models of AD. Therefore, in the final stage of this work, we tested the system PC/Chol/TOC/TPPB-14/DNP as a dosage form for treating mice with AD models. As mentioned above, it has already been shown that free DNP penetrates poorly into the brain via intranasal route. Therefore, at this stage, we assessed the effectiveness of only cationic liposomes.

The experiment on mice with the AD model was performed in two stages: a behavioral “novel object recognition” test and a quantitative assessment of Aβ plaques and the intensity of synaptophysin immunoexpression in the brain of mice. According to the results of the first stage, the use of PC/Chol/TOC/TPPB-14/DNP liposomes allowed for the restoration of the learning ability of the AD model mice (TG+) (57.1 ± 2.8%) almost to the level of healthy wild-type animals (TG−) (69.4 ± 4.3%) (Figure 7). It is worth noting that during the 21-day experimental period, the liposomal drug formulation did not cause any irritation of the nasal mucosa of mice, which is an important criterion in the selection of the drug formulation and its route of administration. The second stage of the experiment was devoted to the quantitative evaluation of Aβ plaques and the intensity of synaptophysin immunoexpression in the brain of mice. The analysis was performed in the entorhinal cortex area of the brain and hippocampus (DG, CA1 and CA3), as these brain regions are responsible for memory formation and impairment. It was demonstrated that the intranasal administration of PC/Chol/TOC/TPPB-14/DNP liposomes for 21 days significantly reduced the percentage of total area of Aβ plaques in the hippocampus and entorhinal cortex of TG+ mice compared to the control group (Figure 8 and Figure S7). It should be noted that the differences between the values for the liposomal drug formulation and the control are statistically significant in all studied areas of the brain except for the area CA3. Thus, the intranasal administration of PC/Chol/TOC/TPPB-14/DNP liposomes for 21 days not only helped to alleviate memory impairment but also influenced the development of AD in transgenic mice by slowing down the rate of Aβ plaque formation.

It should be noted that at the final stage of the study, the level of immunoexpression of synaptophysin (the marker of synaptic contacts) was evaluated, the reduction of which in critical areas of the brain likely leads to memory, cognitive, and behavioral dysfunction. After intranasal administration of the liposomes, the intensity of synaptophysin immunoexpression in all examined areas of the brain was even higher than in the healthy TG− control group (Figure 9). A similar effect has previously been described in the study of antioxidants present in green tea extracts [105]. It can be concluded that the use of PC/Chol/TOC/TPPB-14/DNP liposomes for 21 days has a positive effect on synaptic plasticity.

This study has some limitations, which should be noted. There are general limitations that characterize fundamental research on liposomes, namely, the difficulty in scaling up the production of liposomal formulations and the differences between animal and human models [106,107]. There are also particular limitations for our systems, the elimination of which can be considered as prospects for further development of the work. The enhancement of the mucoadhesive properties of nanocontainers, testing other combinations of drugs that can act on several targets of AD, and the assessment of new surfactants as modifiers can be considered as further steps. In addition, it is important to start treatment with the proposed systems at the early stages of the disease [108]. The most important limitation is the obtainment of approval for biomedical use of the cationic surfactants [109]. However, these limitations do not reduce the importance of our study, as to the best of our knowledge, this is the first study on cationic liposomes with dual substrate loading for intranasal administration.

## 4. Materials and Methods

### 4.1. Objects

Donepezil hydrochloride (DNP, ≥98%, HPLC), cholesterol (Chol, ≥99%), α-tocopherol (TOC), and thioflavin S were procured from Sigma-Aldrich (St. Louis, MO, USA). Soybean L-α-phosphatidylcholine (PC, 95%) (Avanti Polar Lipids, Inc., Alabaster, AL, USA) was used as the main component of liposomes. Tetradecyltriphenylphosphonium bromide (TPPB-14) was synthesized according to the published method [74]. Chloroform and ethanol (HPLC) were purchased from JSC “№1 BASE Chemical reagents” (Staraya Kupavna, Russia). Rhodamine B (RhB) (Acros Organics, Morris Plains, NJ, USA) and fluorescent lipid DOPE-RhB (1,2-dioleoyl-sn-glycero-3-phosphoethanolamine-N-(lissamine rhodamine B sulfonyl) (ammonium salt) (Avanti Polar Lipids, Inc., Alabaster, AL, USA) were used to visualize liposomes inside cells. Liposomal dispersions were prepared using ultrapure Milli-Q water purified by the Simplicity^®^ UV system (Millipore SAS, Molsheim, France). For visualization of the immunoexpression of synaptophysin, primary rabbit monoclonal antibody to synaptophysin (ab 32127) and secondary donkey anti-rabbit (Alexa Fluor^®^ 488) antibody (ab 150073) were purchased from Abcam (Cambridge, UK). Mayer’s hematoxylin was purchased from Biovitrum (Saint Petersburg, Russia).

### 4.2. Liposome Preparation Protocol

To identify the optimal composition of liposomes, the concentration of the components was varied over a wide range: 15 mM, 20 mM, and 30 mM (toward PC/Chol or PC/Chol/TOC) (Table 4). The PC/TPPB-14 ratio in all cases was 50/1. The lipid film hydration method was chosen as a method for the preparation of liposomes. The corresponding weights of lipids were dissolved in 100 µL of chloroform. To include TOC and TPPB-14 in the lipid bilayer, their stock solutions (in ethanol and chloroform, respectively) were prepared and dosed to the lipid mixture to obtain the appropriate concentrations (Table 4). The organic solvents were then evaporated on a rotary evaporator RE-52AA (Shanghai Jingke Scientific Instrument Co., Ltd., Shanghai, China) under vacuum until a lipid film formed. The final film was hydrated with Milli-Q water (in the case of empty liposomes) or an aqueous solution of DNP (in the case of drug-loaded liposomes). In both cases, the liposome dispersions were frozen in liquid nitrogen and thawed in a water bath (5 cycles). The size of the resulting liposomes was controlled by passing the dispersions through a polycarbonate membrane with a pore size of 100 nm using an LiposoFast Basic extruder (Avestin, Ottawa, ON, Canada). Liposomes were stored at 4 °C.

### 4.3. Determination of the Size, Zeta Potential, and Morphology of Liposomes

To control the size, zeta potential, and stability of liposomes, dynamic and electrophoretic light scattering was used. The measurements were carried out on a Zetasizer Nano ZS device (Malvern Instruments Ltd., Worcestershire, UK) at 25 °C. For measurements, all solutions were diluted with Milli-Q water to 2 mM (toward PC). All characteristics of the device and research methods are described in [36].

The size and morphology of the liposomes were confirmed by transmission electron microscopy using a Hitachi HT7700 Exalens microscope (Hitachi High-Technologies Corporation, Tokyo, Japan). For the experiment, fresh dispersions of liposomes were prepared, the concentration of which was carefully selected to achieve an acceptable number of aggregates in the field of view without the formation of a thick film on the grid, i.e., 5 μM. Sample was dispersed on 300 mesh 3 mm copper grid (Ted Pella) with continuous carbon-formvar support films and dried at room temperature. The images were acquired at an accelerating voltage of 100 kV. The calculation of the diameter of the aggregates was carried out using ImageJ software (Version number is 1.53t).

### 4.4. Quantification of Encapsulation Efficiency (EE%)

The methodology for determining the encapsulation efficiency of TOC and DNP differs due to their hydrophobic and hydrophilic nature, respectively. In the case of TOC, the method of extraction of the unencapsulated substrate in ethanol was used [83]. Unencapsulated DNP was separated from liposomes using Amicon^®^ Ultra-0.5 Centrifugal Filter Units (Merck Millipore, Burlington, MA, USA) and Eppendorf MiniSpin microcentrifuge (Eppendorf, Hamburg, Germany). In this case, 0.4 mL of the liposomal dispersion was added to the filter and centrifuged for 10 min at 10,000 rpm. Next, an ethanol solution of TOC and an aqueous solution of DNP from the bottom of the centrifuge filter were diluted to measure absorption spectra on a Specord 250 Plus (Analytik Jena AG, Jena, Germany) using a 0.2 cm quartz cuvette. Encapsulation efficiency was calculated using the following formula:

$$\mathrm{EE}\;\left(\%\right)=\frac{\mathrm{Total}\;\mathrm{amount}\;\mathrm{of}\;\mathrm{substrate}\;-\;\mathrm{free}\;\mathrm{substrate}}{\mathrm{Total}\;\mathrm{amount}\;\mathrm{of}\;\mathrm{substrate}}\times 100\%$$

The experiment was performed at least three times to confirm the validity of the results, which are presented as the mean ± SD.

### 4.5. Quantification of DNP Release Rate In Vitro and Release Kinetic Model Fitting

The release rate of DNP from liposomes (20 mM) was determined by dialysis. Briefly, samples of free DNP and the liposomal form of DNP with a volume of 3 mL were placed in dialysis bags with a pore size of 3.5 kDa (Scienova GmbH, Jena, Germany). Then, the dialysis bags were immersed in beakers containing 60 mL of phosphate-buffered saline (PBS) with a concentration of 0.025 M and pH = 7.4. The concentration of DNP in all samples was 0.5 mg/mL. The DNP release was monitored spectrophotometrically (Specord 250 Plus, Analytik Jena AG, Jena, Germany) at 37 °C with constant stirring (250 rpm). Measurements were conducted using 1 × 1 cm quartz cuvettes. The optical density of DNP was measured from 190 nm to 500 nm, and for calculation of the release rate, the optical density at 317 nm was selected. The experiment was stopped after 24 h, and the optical density at that point was considered to be 100%. The results are presented as the cumulative release percent versus release time. DNP release profiles were fitted to Korsmeyer–Peppas, Higuchi, First-Order, and Zero-Order models using OriginPro 8.5 software according to the following equations in Table 5:

### 4.6. Colocalization

The degree of colocalization of liposomes with mitochondria was determined using a primary culture of rat motoneurons prepared as described by Sibgatullina and Malomouzh [110]. The cells were seeded in 24 × 24 mm glass plates. On the 6th day of cultivation, PC/Chol/TOC and PC/Chol/TOC/TPPB-14 liposomes with DOPE-RhB were added to culture medium and incubated for 24 h. Then, cells were washed twice with PBS and incubated for 20 min in a medium containing MitoTracker Green FM (Thermo Fisher Scientific, Waltham, MA, USA) to stain the mitochondria of the cells. The colocalization degree of liposomes with mitochondria was evaluated using a Leica SP5 TCS confocal scanning microscope (Leica Microsystems, Wetzlar, Germany). DOPE-RhB was excited at 561 nm, and MitoTracker Green FM at 488 nm. The fluorescence emission of DOPE-RhB and MitoTracker Green FM was collected at 570–700 nm and at 500–540 nm, respectively. Pearson’s correlation coefficient was used to identify the dependence between the fluorescence intensities of the two dyes. The validity of the results was checked using the Student’s *t*-test, and *p*-values of less than 0.05 were considered significant.

### 4.7. Antioxidant Activity In Vitro

The antioxidant activity of free TOC (ethanol solution) and TOC-loaded liposomes was determined using an in vitro chemiluminescence assay, in which the intensity of chemiluminescence is a measure of the amount of radicals. Luminol (98%) (Alfa Aesar, Haverhill, MA, USA) was used as a luminophore, the luminescence of which was activated by 2,2′-azobis(2-amidinopropane) dihydrochloride (AAPH, 98%) (Acros Organics, NJ, USA). Before the experiment, a solution of luminol was prepared with a concentration of 1 µM dissolved in 0.1 M NaOH solution. Immediately prior to analysis, the stock solution of luminol was diluted four times with Milli-Q water. For chemiluminescent analysis, 1 mL of the reaction mixture was placed in a cuvette of a Lum-1200 instrument (DISoft, Russian Federation) thermostated at 30 °C. The total volume of the reaction mixture was composed of the following components: 400 µL of 250 µM luminol, 500 µL of 0.1 M TRIS buffer with pH = 8.8 (Fisher Chemical, Waltham, MA, USA), and 100 µL of 40 mM AAPH aqueous solution. Then, the baseline chemiluminescence level was measured for 20 min, after which 10 µL of the test compound was added to the cuvette with the reaction mixture, and the chemiluminescence level was measured. The results obtained were expressed in % toward the initial baseline chemiluminescence level, which was taken as 100%. The results were processed using the PowerGraph and OriginPro 8.5 software.

### 4.8. Animals

In vivo experiments involving animals were carried out in accordance with the Directive of the Council of the European Union 2010/63/EU. The protocol of experiments was approved by the Animal Care and Use Committee of FRC Kazan Scientific Center of RAS (protocol No. 2 from 9 June 2022). Animals were kept in well-ventilated rooms at 20–22 °C in a 12-h light/dark cycle, 60–70% relative humidity. Wistar rats were purchased from the Laboratory Animal Breeding Facility (Branch of Shemyakin-Ovchinnikov Institute of Bioorganic Chemistry, Puschino, Moscow Region, Russia). Transgenic mice with a model of AD were purchased from the Institute of Physiologically Active Substances, Federal Research Center of Problem of Chemical Physics and Medicinal Chemistry RAS (Chernogolovka, Moscow region, Russia).

### 4.9. Visualization of Liposomes into the Rat Brain

To visualize liposomes in the brain, free RhB and RhB in PC/Chol/TPPB-14 (15 mM) liposomes were administered intranasally at a dose of 0.5 mg/kg (400 µL per rat) to Wistar rats. One hour after administration of the liposomal dispersion, animals were euthanized with isoflurane, transcardially perfused with 300 mL of cold PBS (pH = 7.4). Rat brains were removed and frozen in liquid nitrogen. The obtained samples were stored at –80 °C. Twenty-four hours before the experiment, samples were moved to a −20 °C freezer.

For microscopic imaging, samples were cut into 10 µm sections using a Tissue-Tek Cryo3 microtome (Sakura Finetek, Torrance, CA, USA). RhB fluorescence in the brain of rats was observed on a Leica TSC SP5 MP confocal laser scanning microscope (Leica Microsystems, Wetzlar, Germany) using a Cyanine 3 filter at λ_ex_ = 550 nm and λ_em_ = 570 nm. Non-treated animals were used as a control.

### 4.10. Novel Object Recognition Test

The experiments were carried out on transgenic mice of both sexes weighing 24–25 g expressing a chimeric mouse/human protein—a precursor of amyloid beta and a mutant human presenelin-1 (line B6C3-Tg(APP695)85Dbo (APP/PS1)). Liposomes were administered intranasally at 50 µL/mouse for 21 days at a DNP concentration of 1 mg/kg. The control group of animals was administered with an equivalent amount of water. To determine the effectiveness of the proposed therapy in mice with a model of AD, a novel object recognition test was carried out on the 19th day of the therapy [111]. During the test, liposomes were administered 20 min before the start. On the first day, the mice were placed individually into the square testing arena with black walls (50 cm in length, 50 cm in width, 38 cm in height) for 5 min without any objects. On the second day, two identical objects were placed in the central part of the arena, and the mice were allowed to explore the objects for 10 min. On the third day, the mice were presented with a familiar and a novel object for 10 min. The time of exploration of each object by the mice was recorded using a digital camera. After each test, the arena was cleaned with a solution of 70% ethanol. At the end of the test, the preference index (exploration of novel object/total exploration time × 100) was calculated.

### 4.11. Thioflavin S Staining and Immunohistochemistry

On the 21st day of liposomal therapy, mice were anesthetized with isoflurane, transcardially perfused with 30 mL of PBS (pH = 7.4) and then with 4% paraformaldehyde in PBS. After decapitation, the brain was removed, kept for 24 h in a 4% paraformaldehyde solution, and transferred to a 30% sucrose solution in PBS containing 0.02% sodium azide. The brain hemispheres were frozen in a Neg 50 embedding medium, and frontal sections were made with a thickness of 20 µm on a Tissue-Tek Cryo3 microtome (Sakura Finetek, Torrance, CA, USA). To visualize Aβ plaques, brain samples were stained for 5 min with Mayer’s hematoxylin solution (Biovitrum, Saint Petersburg, Russia) and then for 5 min with a 1% solution of Thioflavin S diluted in 50% ethanol. The number and area of Aβ plaques were counted using a LeicaDM 6000 CFS confocal scanning microscope (Leica Microsystems, Wetzlar, Germany). Data analysis was carried out in the entorhinal cortex and hippocampus at ×10 magnification. The results were averaged over 10 sections of the brain of each animal.

To assess the intensity of synaptophysin immunoexpression, the resulting brain sections were incubated in PBS containing 0.1% Triton X-100, 1% BSA, and 1.5% normal donkey serum for 30 min. Next, the sections were transferred to a solution (1:500) of primary rabbit monoclonal antibody to synaptophysin (ab 32127, Abcam) and incubated for 12 h at 4 °C, then washed with PBS and incubated in a solution (1:200) of secondary donkey anti-rabbit (Alexa Fluor^®^ 488) antibodies (ab 150073, Abcam) for 1.5 h at room temperature in the dark. The intensity of synaptophysin immunoexpression was analysed using a LeicaDM 6000 CFS confocal scanning microscope (Leica Microsystems, Wetzlar, Germany). Data analysis was carried out in the entorhinal cortex and hippocampus at ×10 magnification. The results were averaged over 8 sections of the brain of each animal.

### 4.12. Statistics

All data processing was performed using Microsoft Excel 2016^®^ and OriginPro 8.5. Results are expressed as the mean ± standard deviation. Statistical analysis of the results of in vivo experiments (determination of the number of Aβ plaques and intensity of synaptophysin immunoexpression) was carried out using the Mann–Whitney test. ANOVA statistics with Tukey’s post hoc test were used to analyze the results of the behavioral test and DLS data. The validity of the colocalization results was checked using the Student’s *t*-test. Significance was tested at the 0.05 level of probability (*p*).

## 5. Conclusions

For AD treatment, a protocol for the obtainment of new multitargeted lipid carriers by modifying liposomes with tetradecyltriphenylphosphonium bromide and dual loading of substrates (α-tocopherol and donepezil hydrochloride) for intranasal administration was developed. It was shown that cationic liposomes have a high encapsulation efficiency of donepezil hydrochloride, as well as a significant antioxidant activity. From the Korsmeyer–Peppas model, it was confirmed that the release of donepezil is based on Fickian diffusion. The mitotropic activity of the liposomes was investigated on a culture of rat motoneurons using a confocal microscope. The modified liposomes showed significantly better colocalization with the mitochondria of neuronal cells compared with unmodified ones. Photographs of rat brain slices confirm the penetration of modified fluorescently labeled nanocarriers into the brain in vivo. Studies using transgenic mice with an AD model showed that the intranasal administration of liposomes for 21 days reduces the average number of Aβ plaques in the entorhinal cortex from 5.86 ± 0.46 in TG+ mice in the control group to 3.72 ± 0.25 (*p* = 0.00013) in TG+ mice treated with modified liposomes, and their area from 0.12 ± 0.01% to 0.07 ± 0.01% (*p* = 0.0008), respectively. A downtrend in the average number and total area of Aβ plaques was also observed in the dentate gyrus and CA1 region of the hippocampus. Thus, the intranasal administration of modified liposomes made it possible to decrease memory impairment and to influence the development of AD in transgenic mice by slowing down the rate of Aβ plaque formation.

## Figures and Tables

**Table 1:** Physicochemical parameters of liposomes modified with TPPB-14, 4 °C. The data are presented as mean values ± SD. *—the difference with regard to a similar system without TOC is statistically significant at *p* ≤ 0.05; ** at *p* ≤ 0.01; *** at *p* ≤ 0.001. ^#^—the differences from the PdI values on the first day are statistically significant at *p* ≤ 0.05; ^##^ at *p* ≤ 0.01; ^###^ at *p* ≤ 0.001. Statistical analysis was performed using one-way ANOVA test.

System	D_h_, nm	PdI	ζ, mV	D_h_, nm	PdI	ζ, mV
	1st Day			5th Month		
15 mM						
PC/Chol	107 ± 2	0.071 ± 0.027	−9.2 ± 1.2	116 ± 4	0.116 ± 0.005 ^#^	−10.6 ± 1.7
PC/Chol/TPPB-14	105 ± 2	0.109 ± 0.010	8.2 ± 1.3	115 ± 3	0.114 ± 0.014	24.3 ± 1.9
PC/Chol/TOC	105 ± 2	0.067 ± 0.012	−1.8 ± 0.8 ***	114 ± 3	0.115 ± 0.004 ^##^	−17.0 ± 1.4 **
PC/Chol/TOC/TPPB-14	110 ± 3	0.133 ± 0.011	6.8 ± 0.4	121 ± 2	0.101 ± 0.010 ^#^	22.7 ± 3.4
20 mM						
PC/Chol	108 ± 2	0.078 ± 0.006	−7.6 ± 0.5	116 ± 2	0.085 ± 0.008	−1.6 ± 0.5
PC/Chol/TPPB-14	110 ± 2	0.193 ± 0.010	14.3 ± 2.2	115 ± 1	0.114 ± 0.013 ^##^	36.6 ± 0.5
PC/Chol/TOC	104 ± 3	0.071 ± 0.01	−2.9 ± 1.4 **	116 ± 2	0.101 ± 0.005 ^##^	−7.9 ± 1.4 **
PC/Chol/TOC/TPPB-14	113 ± 2	0.231 ± 0.015	11.0 ± 1.0	122 ± 1 ***	0.105 ± 0.008 ^###^	30.5 ± 0.3 ***
30 mM						
PC/Chol	115 ± 2	0.067 ± 0.015	−4.9 ± 0.5	121 ± 1	0.083 ± 0.012	−5.0 ± 0.9
PC/Chol/TPPB-14	115 ± 2	0.120 ± 0.01	23.9 ± 3.4	126 ± 1	0.142 ± 0.029	35.1 ± 1.4
PC/Chol/TOC	103 ± 2 **	0.077 ± 0.005	−3.6 ± 0.7 *	158 ± 1 ***	0.147 ± 0.016 ^##^	−4.0 ± 0.2
PC/Chol/TOC/TPPB-14	110 ± 1 *	0.148 ± 0.017	11.3 ± 2.8 **	120 ± 1 **	0.103 ± 0.021 ^#^	27.3 ± 0.9 **

**Table 2:** The release kinetic model fitting parameters of DNP.

System	Model								
	Korsmeyer–Peppas			First-Order		Higuchi		Zero-Order	
	*n*	k_KP_, %/min^n^	R^2^	k_1_, 1/min	R^2^	k_H_, %/min^1/2^	R^2^	k_0_, %/min	R^2^
DNP	0.299	15.878	0.9680	0.00987	0.9591	5.139	0.8077	0.267	-
PC/Chol *	0.352	10.210	0.9926	0.00602	0.8953	4.431	0.9198	0.233	0.0729
PC/Chol/TPPB-14 *	0.364	9.777	0.9923	0.00641	0.9291	4.562	0.9342	0.240	0.1441
PC/Chol/TOC *	0.381	8.385	0.9961	0.00547	0.9120	4.305	0.9542	0.227	0.2376
PC/Chol/TOC/TPPB-14 *	0.396	7.269	0.9972	0.0047	0.8967	4.047	0.9665	0.214	0.3071

* Total lipid concentration is 20 mM.

**Table 3:** TAR (total antioxidant reactivity) and TRAP (total reactive antioxidant potential) values for pure TOC and its liposomal form (in both cases, the concentration of TOC is 2 mM) obtained by luminol-induced chemiluminescence.

System	Values Received from Chemiluminescence Curves	
	TAR, %	TRAP, s
TOC	36.4 ± 2.9	221 ± 25
PC/Chol/TOC/TPPB-14 *	98.2 ± 0.1	Over 30,000

* Total lipid concentration is 20 mM.

**Table 4:** The concentrations of the components of unmodified and modified liposomes (15, 20, and 30 mM toward PC/Chol or PC/Chol/TOC).

System	PC, mM	Chol, mM	TOC, mM	TPPB-14, mM
15 mM				
PC/Chol	12	3	-	-
PC/Chol/TPPB-14	12	3	-	0.24
PC/Chol/TOC	12	1.5	1.5	-
PC/Chol/TOC/TPPB-14	12	1.5	1.5	0.24
20 mM				
PC/Chol	16	4	-	-
PC/Chol/TPPB-14	16	4	-	0.32
PC/Chol/TOC	16	2	2	-
PC/Chol/TOC/TPPB-14	16	2	2	0.32
30 mM				
PC/Chol	24	6	-	-
PC/Chol/TPPB-14	24	6	-	0.48
PC/Chol/TOC	24	3	3	-
PC/Chol/TOC/TPPB-14	24	3	3	0.48

**Table 5:** Equations for kinetic models of substrate release.

Kinetic Model	Equation
Korsmeyer-Peppas	Q_t_ = k_KP_ ∙ t^n^
Higuchi	Q_t_ = k_H_ ∙ t^1/2^
First-Order	Q_t_ = Q_∞_ ∙ (1 − e^−k^_1_^t^)
Zero-Order	Q_t_ = k_0_ ∙ t

Where Q_t_ is a fraction of drug released in time t; Q_∞_ is the total fraction of drug released; k_KP_ is the release constant taking into account the structural and geometric characteristics of the dosage form, %/min^n^; n is the diffusion release exponent; k_H_ is the Higuchi release constant, %/min^1/2^; k_1_ is the first-order release constant, 1/min; k_0_ is the zero-order release constant, %/min.

## Data Availability

Analyzed data are included in this manuscript. Raw data are available from the authors upon request.

## Supplementary Materials

The following supporting information can be downloaded at: https://www.mdpi.com/article/10.3390/ijms241310494/s1.
