# Supplementary material for: Mitochondria-Targeted Delivery Strategy of Dual-Loaded Liposomes for Alzheimer’s Disease Therapy
Source: Int J Mol Sci. 2023 Jun 22;24(13):10494. doi: 10.3390/ijms241310494 (PMC10341710; doi:10.3390/ijms241310494)
Supplement: Supplementary file 1 [file ijms-24-10494-s001.zip › Supplementary_revised.pdf]

Mitochondria-Targeted Delivery Strategy of Dual-Loaded Liposomes for Alzheimer's Disease Therapy

Leysan Vasileva[1], Gulnara Gaynanova[1], Farida Valeeva[1], Grigory Belyaev[1], Irina Zueva[1], Kseniya Bushmeleva[1], Guzel Sibgatullina[2], Dmitry Samigullin[2,3], Alexandra Vyshtakalyuk[1], Konstantin Petrov[1], Lucia Zakharova[1] and Oleg Sinyashin[1,*]

[1] Arbuzov Institute of Organic and Physical Chemistry, FRC Kazan Scientific Center, Russian Academy of Sciences, 8 Arbuzov str., Kazan, 420088, Russia

[2] Kazan Institute of Biochemistry and Biophysics, FRC Kazan Scientific Center, Russian Academy of Sciences, 2/31 Lobachevsky str., Kazan, 420111, Russia

[3] Kazan National Research Technical University named after A.N. Tupolev - KAI, 10 K. Marx St., Kazan, 420111, Russia

* Correspondence: oleg@iopc.ru

Table S1. Physicochemical parameters of liposomes modified with TPPB-14. D_h – hydrodynamic diameter, PdI – polydispersity index, ζ – zeta potential, 4°C.

Storage time	PC/Chol			PC/Chol/TPPB-14			PC/Chol/TOC			PC/Chol/TOC/TPPB-14		
	D_h, nm	PdI	ζ, mV	D_h, nm	PdI	ζ, mV	D_h, nm	PdI	ζ, mV	D_h, nm	PdI	ζ, mV
**15 mM**												
1 day	107 ± 2	0.071 ± 0.027	-9.2 ± 1.2	105 ± 2	0.109 ± 0.010	8.2 ± 1.3	105 ± 2	0.067 ± 0.012	-1.8 ± 0.8	110 ± 3	0.133 ± 0.011	6.8 ± 0.4
1 week	116 ± 3	0.119 ± 0.001	-12.3 ± 2.3	166 ± 2	0.234 ± 0.008	18.5 ± 1.9	130 ± 8	0.226 ± 0.015	-7.1 ± 0.4	129 ± 3	0.209 ± 0.014	19.0 ± 2.6
2 months	112 ± 2	0.097 ± 0.012	-10.4 ± 0.7	113 ± 3	0.123 ± 0.014	22.5 ± 1.5	111 ± 2	0.130 ± 0.012	-23.1 ± 1.2	115 ± 3	0.140 ± 0.011	22.1 ± 3.6
4 months	114 ± 1	0.188 ± 0.009	-10.3 ± 0.5	116 ± 1	0.124 ± 0.006	20.9 ± 1.3	113 ± 1	0.124 ± 0.024	-13.2 ± 0.3	119 ± 1	0.122 ± 0.010	20.6 ± 0.5
5 months	116 ± 4	0.116 ± 0.005	-10.6 ± 1.7	115 ± 3	0.114 ± 0.014	24.3 ± 1.9	114 ± 3	0.115 ± 0.004	-17.0 ± 1.4	121 ± 2	0.101 ± 0.010	22.7 ± 3.4
**20 mM**												
1 day	108 ± 2	0.078 ± 0.006	-7.6 ± 0.5	110 ± 2	0.193 ± 0.010	14.3 ± 2.2	104 ± 3	0.071 ± 0.01	-2.9 ± 1.4	113 ± 2	0.231 ± 0.015	11.0 ± 1.0
1 week	112 ± 1	0.079 ± 0.028	-2.3 ± 0.5	118 ± 2	0.168 ± 0.00	24.8 ± 2.2	114 ± 1	0.154 ± 0.016	-1.9 ± 0.5	114 ± 1	0.119 ± 0.018	32.3 ± 0.1
2 months	127 ± 2	0.198 ± 0.017	-3.9 ± 0.2	129 ± 5	0.202 ± 0.024	40.5 ± 4.3	108 ± 2	0.074 ± 0.015	-8.4 ± 1.2	119 ± 4	0.155 ± 0.025	34.8 ± 1.5
4 months	112 ± 1	0.092 ± 0.017	-6.4 ± 1.9	114 ± 1	0.150 ± 0.003	35.9 ± 1.4	113 ± 1	0.088 ± 0.018	-11.2 ± 3.3	130 ± 2	0.186 ± 0.005	27.0 ± 1.0
5 months	116 ± 2	0.085 ± 0.008	-1.6 ± 0.5	115 ± 1	0.114 ± 0.013	36.6 ± 0.5	116 ± 2	0.101 ± 0.005	-7.9 ± 1.4	122 ± 1	0.105 ± 0.008	30.5 ± 0.3
**30 mM**												
1 day	115 ± 2	0.067 ± 0.015	-4.9 ± 0.5	115 ± 2	0.120 ± 0.01	23.9 ± 3.4	103 ± 2	0.077 ± 0.005	-3.6 ± 0.7	110 ± 1	0.148 ± 0.017	11.3 ± 2.8
1 week	115 ± 1	0.058 ± 0.039	-5.5 ± 1.7	116 ± 1	0.103 ± 0.011	25.5 ± 1.8	105 ± 2	0.071 ± 0.017	-5.8 ± 0.4	112 ± 2	0.141 ± 0.060	23.5 ± 2.1
2 months	124 ± 1	0.127 ± 0.020	-6.9 ± 1.2	120 ± 4	0.140 ± 0.023	41.1 ± 3.3	111 ± 4	0.138 ± 0.024	-7.2 ± 1.2	112 ± 2	0.125 ± 0.018	31.9 ± 2.2
4 months	121 ± 1	0.104 ± 0.023	-14.4 ± 8.3	123 ± 2	0.121 ± 0.019	35.3 ± 0.9	126 ± 3	0.154 ± 0.023	-7.2 ± 0.5	117 ± 1	0.121 ± 0.013	26.3 ± 2.9
5 months	121 ± 1	0.083 ± 0.012	-5.0 ± 0.9	126 ± 1	0.142 ± 0.029	35.1 ± 1.4	158 ± 1	0.147 ± 0.016	-4.0 ± 0.2	120 ± 1	0.103 ± 0.021	27.3 ± 0.9

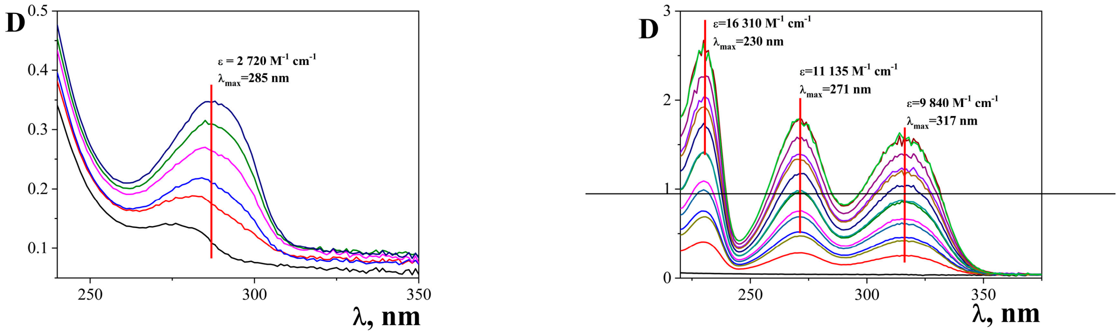

(a) **(b)**

Figure S1. Absorption spectra and extinction coefficient of (a) α-tocopherol (in ethanol) and (b) donepezil hydrochloride (in Milli-Q water); cuvette thickness = 1 cm.

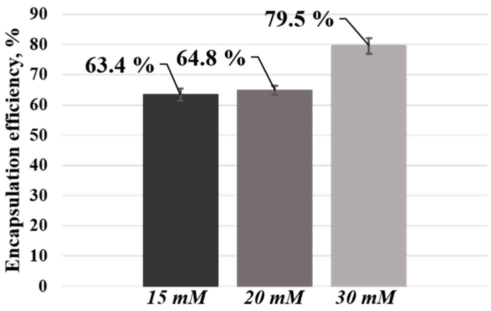

Figure S2. Encapsulation efficiency of PC/Chol/TPPB-14 liposomes toward donepezil hydrochloride.

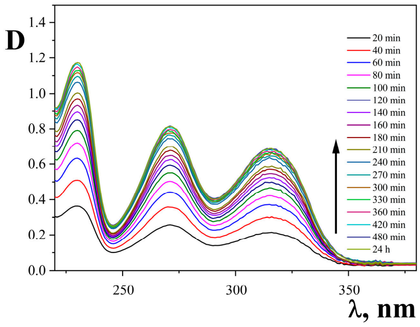

(a)

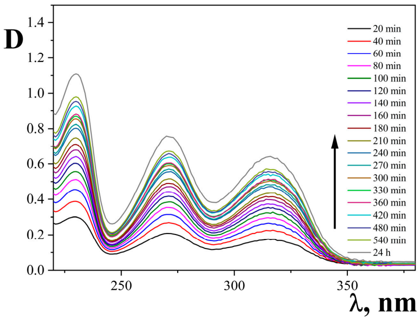

(b)

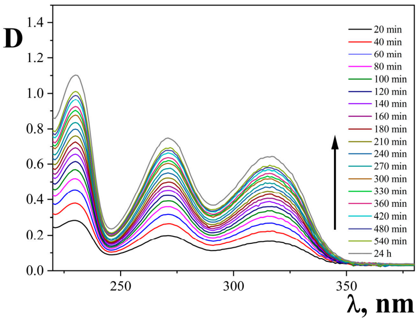

(c)

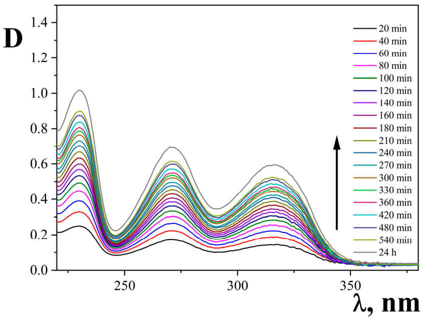

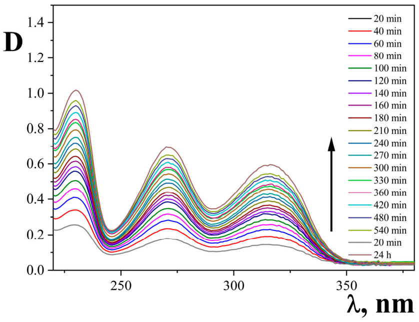

(d) (e)

Figure S3. The absorption spectra of DNP at different time intervals of release for: (a) free DNP; (b) PC/Chol/DNP; (c) PC/Chol/TPPB-14/DNP; (d) PC/Chol/TOC/DNP; (e) PC/Chol/TOC/TPPB-14/DNP. Total lipid concentration is 20 mM. Phosphate buffer (0.025 M), pH = 7.4, 37°C; cuvette thickness = 1 cm; the arrow indicates the direction of dialysis duration increasing.

Table S2. The release kinetic model fitting parameters of DNP*.

**System**	**Model**								
	**Korsmeyer-Peppas**			**First-Order**		**Higuchi**		**Zero-Order**	
	n	k_{KP}, %/min^n	R^2	k_1, 1/min	R^2	k_H, %/min$^{1/2}$	R^2	k_0, %/min	R^2
**DNP**	0.392	10.234	0.9940	0.010	0.9533	5.929	0.9691	0.428	0.4570
**PC/Chol****	0.403	7.977	0.9993	0.007	0.8775	4.881	0.9801	0.354	0.5065
**PC/Chol/TPPB-14****	0.408	7.915	0.9999	0.007	0.8973	4.965	0.9815	0.360	0.5127
**PC/Chol/TOC****	0.415	7.123	0.9992	0.006	0.8778	4.630	0.9848	0.336	0.5358
**PC/Chol/TOC/TPPB-14****	0.417	6.539	0.9991	–	–	4.299	0.9855	0.312	0.5405

* Data obtained for the initial part of the DNP release curves, i.e., from 0 min to 270 min.
** Total lipid concentration is 20 mM.

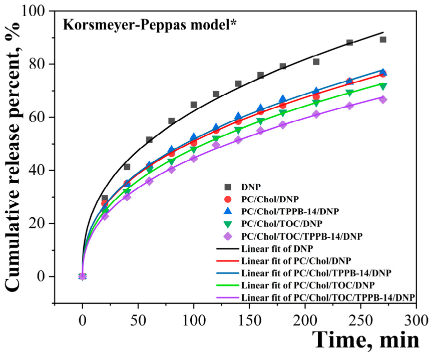

(a)

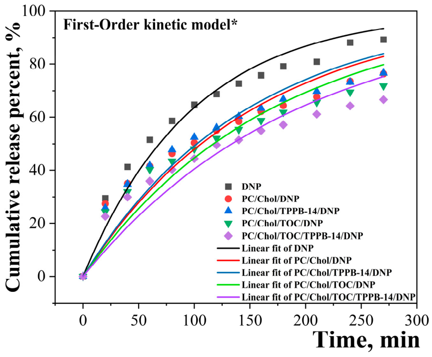

(b)

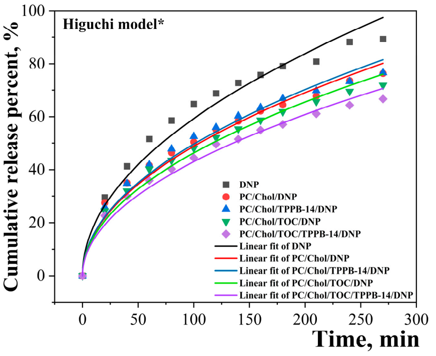

(c)

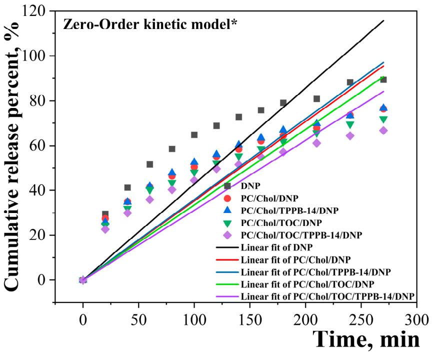

(d)

Figure S4. The release kinetic model fitting curves of DNP: (a) Korsmeyer–Peppas model; (b) First-Order kinetic model; (c) Higuchi model; (d) Zero-Order kinetic model. Total lipid concentration is 20 mM. Phosphate buffer (0.025 M), pH = 7.4, 37°C. * Data obtained for the initial part of the DNP release curves, i.e., from 0 min to 270 min.

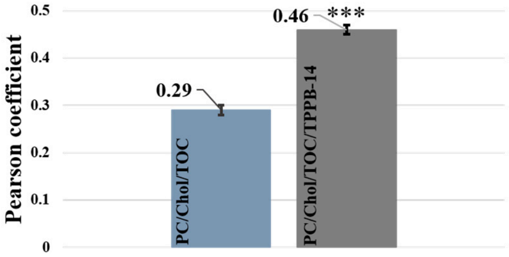

Figure S5. Pearson's Correlation Coefficient for modified and unmodified liposomes. Data are presented as means ± SD. *** — difference with unmodified liposomes is statistically significant at $p \leq 0.001$. Statistical analysis was performed using the One-Way ANOVA test.

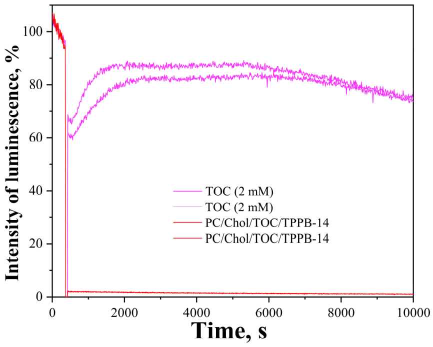

Figure S6. Dependences of chemiluminescence intensity on time. The experiment was carried out twice for each sample.

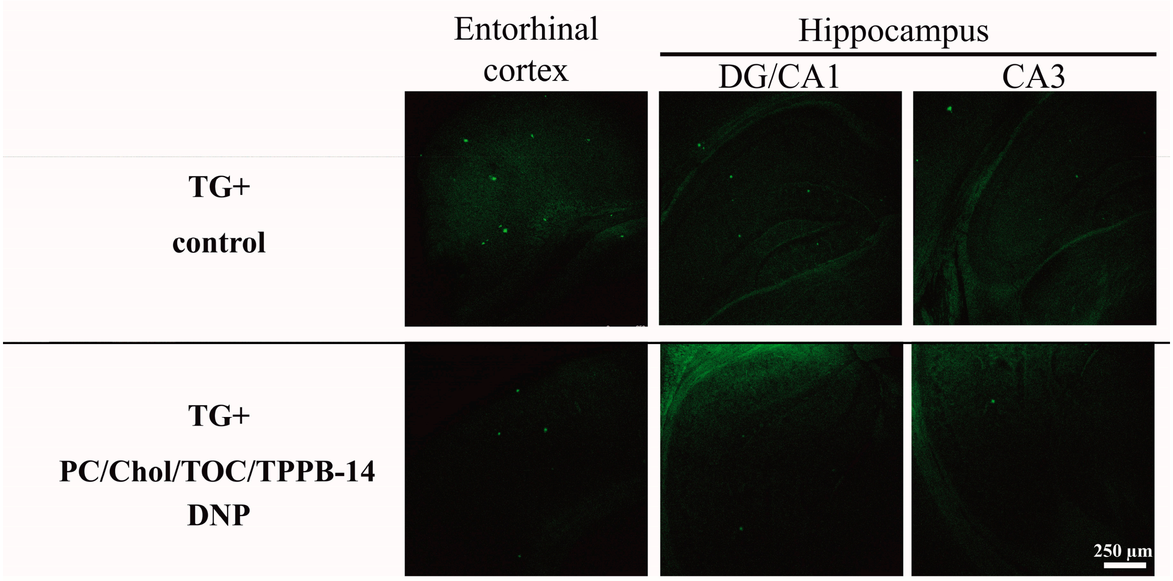

Figure S7. Visualization of Aβ plaques in the entorhinal cortex and areas of hippocampus of control group of transgenic (TG+) mice and group of transgenic (TG+) mice treated with PC/Chol/TOC/TPPB-14/DNP liposomes. Scale bar 250 μm.
